# The acceptability and feasibility of an intercultural birth center in the highlands of Chiapas, Mexico

**DOI:** 10.1186/1471-2393-13-94

**Published:** 2013-04-16

**Authors:** Kathryn Tucker, Hector Ochoa, Rosario Garcia, Kirsty Sievwright, Amy Chambliss, Margaret C Baker

**Affiliations:** 1 Department of International Health, NHS, Georgetown University, Washington, DC 20057, USA; 2 El Colegio de la Frontera Sur, Carretera Panamericana y Periférico Sur s/n, Barrio María Auxiliadora, San Cristóbal de Las Casas, Chiapas CP 29290, México; 3 Department of International Health-NHS, 3700 Reservoir Road, NW, Washington, DC 20057-1107, USA

## Abstract

**Background:**

An intercultural birthing house was established in the Highlands of Chiapas, Mexico, as an intervention to reduce maternal mortality among indigenous women. This birth center, known locally as the *Casa Materna*, is a place where women can come to give birth with their traditional birth attendant. However, three months after opening, no woman had used the birthing house.

**Methods:**

This study reports on the knowledge, attitudes and practices related to childbirth and use of the *Casa Materna* from the perspective of the health workers, traditional birth attendants and the program’s target population. Structured interviews, in-depth interviews and focus group discussions were conducted with participants from each of these groups. Data was searched for emerging themes and coded.

**Results and conclusions:**

Findings show that the potential success of this program is jeopardized by lack of transport and a strong cultural preference for home births. The paper highlights the importance of community participation in planning and implementing such an intervention and of establishing trust and mutual respect among key actors. Recommendations are provided for moving forward the maternal health agenda of indigenous women in Chiapas.

## Background

### Maternal mortality in Chiapas

The indigenous population of Chiapas, México, has a high rate of maternal mortality: one study conducted in indigenous communities in conflict areas in Chiapas found that the maternal mortality ratio was 607/100,000 live births [1], compared to 73/100,000 live births for the state of Chiapas and 60/100,000 live births in all of Mexico in 2009 [2]. The main direct causes of death in Chiapas in 2009 were postpartum hemorrhage followed by hypertensive disorders, reflecting trends in low income countries [3].

Across the globe, the ideal setting for women to deliver is in a health facility, cared for by a skilled birth attendant. However, many women, particularly if they live in rural areas or are members of marginalized ethnic groups, are instead assisted at delivery by traditional birth attendants (TBAs). Births in Chiapas are attended by healthcare professionals in 49.1% of cases, but traditional birth attendants (known locally as ‘*parteras*’) attend the majority of births, especially in areas with a large indigenous and/or rural population [4]. Various explanations for high rate of home births in different settings can be found in the literature including: poor quality of health services being offered, lack of resources at the health centers, and a lack of inter-cultural understanding between the indigenous patients and the mestizo health workers [1,5].

Addressing the issue of improved access to maternal health for indigenous women is an important issue in all of the Americas, where 10% of the populations are reportedly indigenous [6]. While data on maternal mortality rates for this population of about 48 million people is not in systematically collected, there is some evidence that these rates are often two or three times as high as that of the national average. Studies suggest that poor access to health care and preventative services is the norm for the indigenous population of Latin America and that services that do exist are culturally inappropriate [6].

Integration of Western and Indigenous medicine has been shown to result in increased confidence in western health services and to increase access for these vulnerable populations [4,7]. In Mexico there is a legal precedent for appropriate and respectful intercultural health services: the Mexican Constitution recognizes the use of traditional medicine as a cultural right and the Secretary of Health has an obligation to recognize, respect and promote the use of traditional medicine and adapt services to the needs and traditions of the indigenous population. In addition, the National Health Plan 2007–2012 specifically calls for the promotion of intercultural health services and for an increased knowledge of traditional and complementary medicine [8].

In order to address the problem of high maternal mortality in Chiapas, the State of Chiapas Secretary of Health built a birthing house (known as *Casa Materna*, or “Mother’s House”) adjacent to a hospital in the primarily indigenous municipality of San Andrés Larraínzar. This birthing house is intended to look more like indigenous houses than a hospital setting and provides a hygienic environment where women can give birth traditionally with the assistance of their local TBA. Hospital and obstetric staff are easily accessible next door should complications arise during delivery. The TBAs are responsible for referral of patients and for bringing the women to the *Casa Materna*. Thus, a second component to the *Casa Materna* intervention, also provided by the State of Chiapas Secretary of Health, is training of the TBAs. The TBAs continue to be paid directly by the patient and receive no further remuneration from the government [9,10]. It should be noted that the term “*Casa Materna*” has been used to describe other quite different interventions such as *Casa Materna*s in Guatemala which serve as waiting houses, housing women before and after birth [11]. The *Casa Materna* in San Andrés Larraínzar is an independently organized intervention, although there are similarities to other *Casa Materna*s within Chiapas.

It was estimated that the *Casa Materna* could attend about one delivery per day (over 300 in one year) but, when this study was conducted in October and November of 2010, the *Casa Materna* had been open for three months and no woman had yet used it to give birth. By 2012 only five women had given birth in it (*personal communication Hospital Director*). The purpose of this study was to analyze the knowledge, attitudes and practices of those who manage and use the *Casa Materna* in order to provide recommendations to improve maternal health in the region. Findings are also of relevance to other programs seeking to provide maternal health services to indigenous populations.

## Methods

### Population

San Andrés Larráinzar is a municipality in the highlands of Chiapas, Mexico. In 2010, the population was 20,349 and 99.3% of the inhabitants aged three years and more spoke an indigenous language [12]. In 2000, 13.2% of the population lived in an urban setting and the remaining 86.8% lived in one of 61 rural localities. In 2010, 69.6% of the population lived in a dwelling with a dirt floor, and the population older than fifteen years old had an average of five years of schooling [12]. The study was conducted in the capital of the municipality, San Andrés Larráinzar, and Bayalemon, a village 25 minutes driving from San Andrés Larráinzar.

This study used qualitative and quantitative methods to gauge the feasibility and acceptability of the *Casa Materna* program in San Andrés Larráinzar according to the major stakeholders in the program. These were: the health personnel at the adjacent hospital who would attend women coming to the *Casa Materna*; the traditional birth attendants who were intended to act as key intermediaries in the program, bringing the women to the *Casa Materna* and attending them there; and the program target group, the indigenous women from the surrounding communities.

### Data collection methods

Instruments used were: in-depth interviews with seven traditional birth attendants; three focus group discussions (FGD) with women from the community, including individual completion of a short structured interview; structured interviews with eleven health personnel; and the observation of two TBA training sessions. Informed verbal consent was obtained from all participants.

The TBAs were selected from those who regularly attend the training sessions at the *Casa Materna* and were recruited by the coordinators of the training program to participate in the project. The health personnel contacted the TBAs and a convenience sample of those who arrived at the health center was interviewed. The interviews were completed in the village of San Andrés Larráinzar and the community of Bayalemon. The topics covered in the in-depth interviews with the TBAs included: how they learned to be *parteras*, the traditional way to treat patients, barriers to health care for the indigenous population, their experiences in the training course, their relationship with the health personnel, and their opinions about the *Casa Materna*.

The participants for the three focus groups were recruited from women who had given birth to a child in the previous five years and who were visiting the health center in San Andrés Larráinzar (n = 4) and Bayalemon (n = 17) on that day. Focus groups concentrated on three major themes: the women’s experience during pregnancy and childbirth; their perceptions of and experiences in the health services; and their opinions of the program of the *Casa Materna*. The structured questionnaire was administered to each woman who participated in the focus groups and included demographic questions as well as questions about their individual preferences for *parteras* or health service personnel for prenatal care and care during childbirth.

The health personnel interviewed were those who were working on the day selected by the researchers for interviews. They included five doctors, five nurses, and one social worker. The interviews focused on: their perceptions of how well the hospital covers the needs of the indigenous population; barriers to healthcare that the indigenous population faces; their participation in intercultural workshops; their knowledge of traditional practices; their perceptions of TBAs; and their opinions about the *Casa Materna* program.

The first author, who speaks Spanish but not Tzotzil, observed two training sessions. While one training session was held in Spanish and translated to Tzotzil, the researcher was limited in observations during the second training session that was held mainly in Tzotzil.

The first author scheduled focus groups with indigenous men, but the intended participants did come and time constraints prevented more attempts to reach this population.

This study was conducted under the auspices of the El Colegio de la Frontera Sur and according to Mexican regulations it was considered as exempt from IRB review due to the non invasive methods used. Consent was obtained verbally from all participants.

### Data analysis methods

A trained female translator who speaks both Tzotzil and Spanish facilitated the FGDs with the women and in-depth interviews with the TBAs. She audio taped them and then transcribed them in Spanish. The interviews with health personnel were conducted in Spanish by the first author who took detailed notes during and immediately after the interviews. Notes were also taken on the observation of the training sessions.

All qualitative data was analyzed manually in Spanish by grouping the data into themes that emerged during data analysis using grounded theory. Themes referred to barriers to accessing health services, preferences for use of TBAs, relationships between the different actors, perspectives on the *Casa Materna* intervention, and training provided by the intervention. This is used as a framework for presenting the results.

Structured questionnaires administered to the women attending FGDs were analyzed using Excel.

## Results

### Demographics

The seven TBAs who were interviewed had an average of 33 years of experience, with a range of 14 to 50 years of practice.

**Figure 1:**
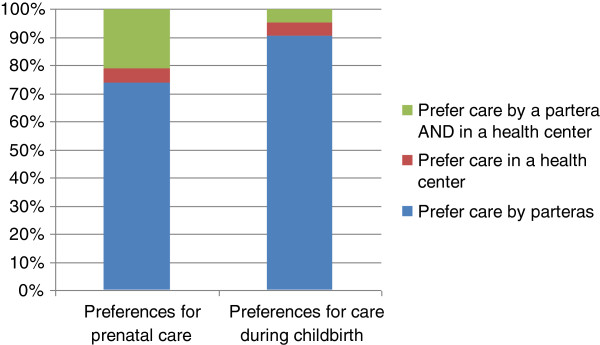
Preferences of women in focus groups for care during pregnancy and childbirth (n = 21).

The women who took part in the FGD had an average age of 27.9 years with an average of 5.3 years of formal schooling; two-thirds of the participants said they can read and write a little and 10% could not read or write at all. Although the focus groups took place in San Andrés Larráinzar and Bayelemón, the women came from surrounding villages. They were on average 17.8 years old when they had their first child, and had an average of 3.4 children; 97.2% of those children were born with the aid of a *partera*. Figure 1 shows the women’s preferences for their next birth.

The eleven health personnel interviewed had worked an average of one and a half years in the hospital, and about half of the personnel had worked there for less than one year. Nine of the health personnel reported working directly in maternal healthcare for indigenous women, one was an administrator involved in the planning of the *Casa Materna*, and the other was a nurse who aids in general consultations and gives vaccinations.

### Perspectives on barriers to health care

**Figure 2:**
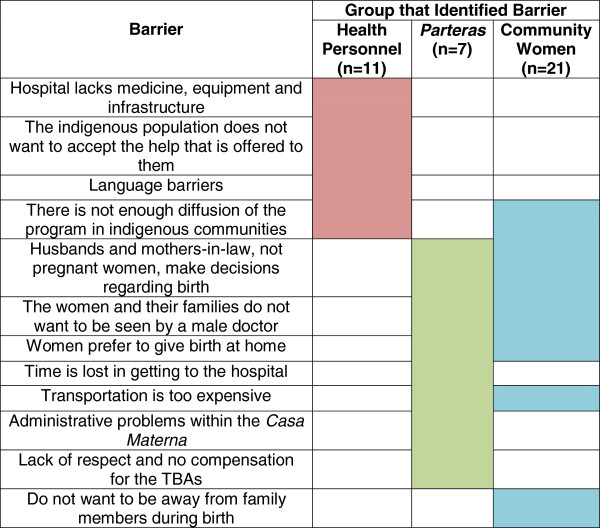
**Summary of barriers to using the *Casa Materna *identified.**

Perceived barriers to using western health services are summarized in Figure 2, highlighting the difference between the perspectives of the different actors. While the health personnel understood the main barriers to be lack of equipment at the hospital and the refusal of the indigenous population to accept the help offered, the women and TBAs from the community focused on physical and cultural barriers.

The hospital is far from many communities and the transportation can be expensive. Cultural and socioeconomic barriers and customs also influence the decision of whether or not to go to the hospital, reinforcing the physical barriers:

*Sometimes I think for the money, for this we stay in the house with the TBAs and we stay closer as well. Because our mothers-in-law also gave birth here, for this reason we stay in the house* (Focus Group 1, Bayalemon, November 4, 2010).

### Preferences for care from TBAs or hospital services

Although the women did not openly object to visiting the health center during pregnancy, there was a strong preference in each of the focus groups for a home birth with a TBA.

In all the focus groups, the women expressed confidence in the work of the TBAs, but acknowledged the complementary use of the formal health sector for care during pregnancy. The women also mentioned that, for both pregnancy and birth, decision-making power resides with the husbands or mothers-in-law, and the pregnant women may not have control over which TBA she sees or whether or not she goes to a health center. It is also important for family members to accompany the woman during birth, which makes going to the hospital a more expensive and less desirable option.

In each focus group, the women expressed satisfaction with the treatment they had received in the hospital or health center. There were also two women who mentioned giving birth in the hospital and both reported that they had been treated well. However, one of the TBAs mentioned a different perspective that may help to explain why women are reluctant to go to the health services for care during childbirth:

*I tell the women to go to the clinic to be attended but they don’t want it…and furthermore they say that they are not going to be cared for as they ought to be because in their houses they are well cared for* (*Partera* 7, Bayalemon, November 5, 2010).

### Relationship between TBAs, indigenous women and health personnel

**Figure 3:**
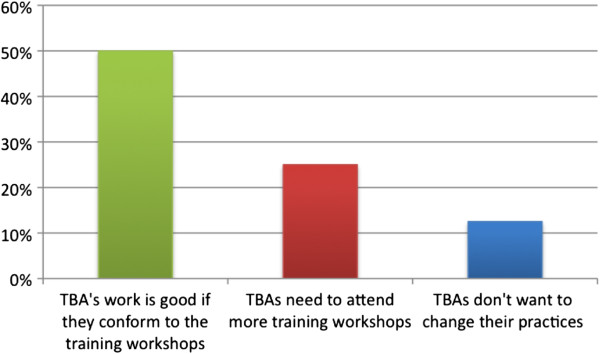
Health personnel opinion of the work of TBAs (N = 11).

The health personnel were asked about their experience working with TBAs in the hospital. 72.3% (n = 8) of the participants have regular contact with traditional birth attendants in their work. When asked their opinion of the work of the TBAs, the answers varied: one of the participants thought that the TBAs are too aggressive in treating childbirth, while another thought that they are warm, caring and understanding with their patients. Figure 3 summarizes the opinions of the health personnel regarding TBAs.

The TBAs view their working relationship with the health services as having two major components: referring women to doctors for risky pregnancies and reporting each month on the pregnancies and births they have attended. The women expressed the desire to continue to work with the health personnel, but one TBA also stressed their independence as healers in their own right. One of the TBAs mentioned that she attends the training sessions because she is worried that she might be blamed for a complication in her client’s pregnancy; this way, she said, the government will protect her in the case of a problem. Whether or not the TBA would get in trouble or the health services would protect her in case of complications is unclear, but this opinion strengthens the observation that the TBAs feel pressured to do what is asked of them by the health services.

All of the TBAs were frustrated by the fact that they are not given travel stipends or recognized by the government, which seems to put a strain on the relationship between TBAs and health personnel:

*What we would like…is that the government recognizes our work, what we do with the pregnant women. We give of our time and nobody recognizes us (Partera* 2, San Andrés Larráinzar, October 29, 2010).

Although the women expressed desire to work with the doctors, this answer demonstrates that the TBAs do not feel respected as partners in this program.

### The *Casa Materna* program

**Figure 4:**
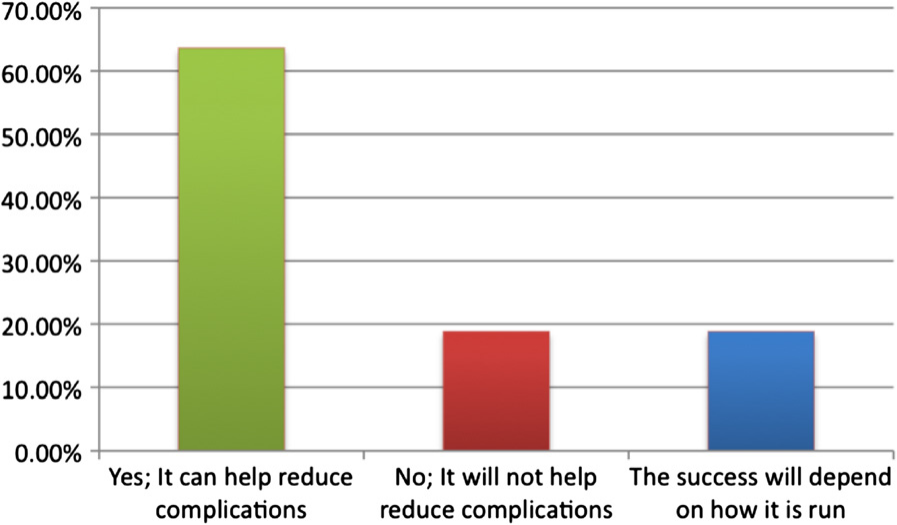
**Opinions of the health personnel as to whether the *Casa Materna *program will be successful? (N = 11).**

The health personnel participants had mixed feelings about the feasibility of this program, which are summarized in Figure 4. Common consensus was that the program was positive and provided a mechanism for closer patient supervision. On the other hand, personnel also mentioned institutional problems such as lack of trained staff and cultural problems such as a lack of acceptance by the indigenous population.

The health personnel provided many recommendations for the program. The most common were to have more promotion of the program in communities, continue training courses for TBAs, provide transportation to the *Casa Materna*, and to have a TBA who works full time in the *Casa Materna.*

One of the health workers mentioned that the health personnel themselves do not have a good understanding of the program and there appeared to be confusion as to whether the program was even operational.

When directly asked all of the TBAs stated that they liked the program of the *Casa Materna*. However, further conversation uncovered issues and misunderstandings about the program. Each of the TBAs emphasized that their patients do not want to participate in the program; furthermore, a few of the TBAs themselves said that they are not willing to come to the Casa to help their patients give birth.

There is evidence that the TBAs and the indigenous community have not been adequately integrated into the planning and implementation of the program. One *partera* mentioned that she came to the *Casa Materna* with a woman in labor, only to find it locked. The *temascal*, or small earthen steam-bathing room essential in traditional treatment during pregnancy and childbirth [13], at the *Casa Materna* is large and igloo-shaped. It does not resemble the traditional small earthen temascales found in the community and the TBAs doubted that it would function well.

When asked, the TBAs did not provide concrete recommendations for the *Casa Materna*. One mentioned that she does not understand the program. Most said that they do not have recommendations because the women simply do not want to come. Another TBA said that only the doctors know what should be done. However, one TBA in Bayalemon mentioned that it might work for people who live close to the hospital and for those who have complicated pregnancies and need to be near the hospital.

The women in the focus groups confirmed what the TBAs said: they do not want to go to the *Casa Materna*. In Bayalemon, none of the women had heard of the *Casa Materna*. The program was explained to them and when asked if they would consider going there to give birth they responded that they prefer to give birth at home with their family and their TBA. Even though Bayalemon is only twenty minutes away from the hospital in San Andrés Larráinzar, neither the TBAs nor the women expressed optimism that women from this community would decide to use the program.

### Training program

All of the TBAs reported learning their skills in specific cultural ways, usually by dreams or revelations. Although four of the TBAs mentioned that their family members had also been *parteras*, they stressed dreams rather than observation as how they learned the practice. The communities accept these cultural forms of learning as valid and begin to request the services of the *partera* after she experiences her dream or revelation.

The training workshops held by the health personnel in the *Casa Materna* assumed a didactic-model of learning and utilized PowerPoint presentations to explain biomedical subjects. In the translated session, the TBAs remained silent throughout most of the presentation; they only asked a few questions and were not asked to participate. In the Tzotzil session, the women were more animated and engaged in the discussion.

When asked directly, all of the TBAs responded that they like the training courses that they attend in the *Casa Materna*, and spoke well of those who offer it. However, there were other indications in the interviews that there are problems with the workshops. One participant, for example, feels like the themes for each month are always the same. There is also evidence that the TBAs feel that the doctors do not respect their time or other responsibilities. Money and lost time were mentioned by almost all of the TBAs as barriers to attending the courses. They also mentioned that they are not reimbursed for their transportation fares and often are not able to eat all day because they are away from home.

Many of the health personnel in the hospital are working there as part of the ‘social service’ that is required of all graduates in Mexico, so they do not come from the area or know the local culture. The health personnel also noted that there is little opportunity for them to learn about the culture of the communities they serve. Of the health personnel interviewed, 45.4% had participated in the past 5 years in an ‘intercultural workshop,’ where they learned about different cultures. The efficacy of the workshops as well as the topics they covered varied and was difficult to gauge from their answers. It is significant to note that the two participants who work specifically in the *Casa Materna* and help to run the TBA trainings have never attended an intercultural workshop. Although the majority of the health personnel expressed an interest in learning more about the community they served, there was no such training offered to them at the hospital.

## Discussion

All of the participants in this study mentioned significant cultural and socioeconomic barriers that the indigenous population faces in seeking healthcare. However, there was a significant discrepancy between the barriers mentioned by health personnel and those mentioned by the indigenous women and their TBAs: the health personnel focused on institutional problems with the hospital itself while the indigenous population drew attention to geographical barriers and cultural preferences (Figure 2). This discrepancy illustrates a lack of understanding between the health personnel and indigenous population.

The women in this study are willing to go to health centers for prenatal care but, they strongly prefer to give birth at home. This finding is supported by a study conducted by Sosa-Rubi *et al.* which uses regression analysis to study data collected by the Rural Evaluation Survey designed to evaluate Mexicos’ Oportunidades program. Sosa-Rubi and her colleagues observe that although the Oportunidades program has influenced women’s selection towards health facility delivery in rural areas, this is not the case among indigenous women. They also noted that choice of delivery location was not associated with the health center’s quality index [14].

The data presented in this paper shows that the birth preferences of the indigenous population are formed by their culture. Strong familiar and cultural forces exist favoring a home birth with a *partera.* The woman and her family must choose between a home birth surrounded by family with a *partera* who speaks her language and understands her culture, or paying to go somewhere else where these comforts may be diminished or nonexistent. The design of the *Casa Materna* intervention focuses on improving the supply of maternal services but it does not address the lack of demand for these services. The strong cultural preference for a home birth is not taken into consideration, nor is there an appreciation for the decision facing the family who has always depended on the wisdom of a *partera* during birth.

This study found no culture of collaboration between the health personnel and the TBAs, and no apparent precedent for intercultural exchange. The health personnel had received little intercultural training and there was no evidence of community participation in the planning and implementation of the program. All of the participants identified lack of diffusion as a major barrier to the success of the program. The *Casa Materna* relies on the diffusion of the program from the TBAs to their patients, but the TBAs were not invested in the success of the program nor did they feel like partners in the process. In addition, it is the husband or mother-in-law, not the woman or the TBA, who decides where a woman will give birth, and there was no evidence of community outreach to either of those two populations.

Interventions delivered solely by health personnel without a community base have been demonstrated to be largely unsuccessful [15]. On the other hand, efforts to integrate Western and Indigenous medicine can result in greater access for vulnerable populations and more confidence in Western health services [4,7]. Intercultural health services have been shown to be successful when there is a mutual respect between the practitioners of both traditional and biomedical ‘medical’ systems [7,16,17]. This requires an exchange of information about practices and beliefs so that TBAs and health personnel teach and learn from each other. It is also essential to have the target population involved in the planning, execution and evaluation of the project. A TBA training program in Guatemala found that respecting TBAs as authoritative sources of knowledge and incorporating traditional practices and beliefs into training sessions improved the acceptability of the training sessions. In addition, a non-hierarchical structure was established between biomedical personnel and TBAs based on mutual trust [18]. A review of intercultural health programs in Guatemala, Suriname, Chile, Colombia and Ecuador also found that collaboration, respect for indigenous practices, and integration of the indigenous community into the health centers are essential for success and acceptance of the program [19].

## Conclusions

The results of this study cast into doubt the feasibility and acceptability of the *Casa Materna* intervention in its current form because of the cultural prerogative for home birth and the TBAs’ and community’s lack of engagement in the design and investment in the implementation of the program. The program was three months old at the start of the study, and nobody had used the *Casa Materna* for birth; the only TBA interviewed who had tried to use the program found the Casa locked.

The following recommendations are made to improve the feasibility and acceptability of the *Casa Materna* program: a committee of TBAs, health personnel and community members (both male and female) should be created to develop a culturally acceptable model of care, including deciding the fate of the *Casa Materna;* the *parteras* should be given some formal recognition and financial aid to offset the costs of attending the training program; the training programs should be re-conceptualized to promote intercultural communication; and the program needs to be effectively diffused to the surrounding communities.

It is important to note that the most significant barrier to the success of the program is the cultural prerogative for home births. A solution will only be found through sincere and respectful communication between the health personnel and the indigenous community.

### Study limitations

The TBAs and indigenous women were either recruited by health personnel or chosen from patients in a health center. Because of this, it is plausible that the investigators were perceived to be from the health services. It is also possible that the perspectives of women attending health centers are different to those who do not use health services. However in both cases one would expect any bias of results to tend towards being positive about health services. While this study reported few positive responses on use of health services it is possible that stronger negative perspectives were missed. .

The lack of focus groups with men and older women means that the opinions of those who decide where a woman gives birth were not heard. A study from Mexico has shown that women typically lack autonomy and need to receive permission from their partners to seek care [20]. The researchers also reported that many women did not receive care from maternal health services because “men expressed jealousy if their wives were examined by a health practitioner”. However, the literature on the perspectives of men is very sparse and remains a critical topic for future research and for consideration in the planning of interventions.

## Competing interests

The authors declare that they have no competing interests.

## Authors’ contributions

KT carried out the research in the field, conducted the data analysis and drafted the manuscript. HO and RG participated in the design of the study, it’s coordination, and interpretation of findings. KS and AC participated in literature reviews, interpretation of findings and contributed to writing the manuscript. MB contributed to interpretation of findings and helped draft the manuscript. All authors read and approved the final manuscript.

## Pre-publication history

The pre-publication history for this paper can be accessed here:

http://www.biomedcentral.com/1471-2393/13/94/prepub
